# Inorganic Nanocarriers Overcoming Multidrug Resistance for Cancer Theranostics

**DOI:** 10.1002/advs.201600134

**Published:** 2016-05-30

**Authors:** Gan Lin, Peng Mi, Chengchao Chu, Jun Zhang, Gang Liu

**Affiliations:** ^1^ State Key Laboratory of Molecular Vaccinology and Molecular Diagnostics & Center for Molecular Imaging and Translational MedicineSchool of Public HealthXiamen UniversityXiamen361102China; ^2^ Department of Chemical and Biomolecular EngineeringThe University of MelbourneParkvilleVictoria3010Australia; ^3^ State Key Laboratory of Biotherapy and Cancer Center West China Hospital Sichuan University, and Collaborative Innovation Center for BiotherapyChengduSichuan610041China; ^4^ Department of UltrasoundXijing HospitalXi'anShaanXi710032China

**Keywords:** inorganic nanocarrier, molecular imaging, multidrug resistance, theranostics, cancer

## Abstract

Cancer multidrug resistance (MDR) could lead to therapeutic failure of chemotherapy and radiotherapy, and has become one of the main obstacles to successful cancer treatment. Some advanced drug delivery platforms, such as inorganic nanocarriers, demonstrate a high potential for cancer theranostic to overcome the cancer‐specific limitation of conventional low‐molecular‐weight anticancer agents and imaging probes. Specifically, it could achieve synergetic therapeutic effects, demonstrating stronger killing effects to MDR cancer cells by combining the inorganic nanocarriers with other treatment manners, such as RNA interference and thermal therapy. Moreover, the inorganic nanocarriers could provide imaging functions to help monitor treatment responses, e.g., drug resistance and therapeutic effects, as well as analyze the mechanism of MDR by molecular imaging modalities. In this review, the mechanisms involved in cancer MDR and recent advances of applying inorganic nanocarriers for MDR cancer imaging and therapy are summarized. The inorganic nanocarriers may circumvent cancer MDR for effective therapy and provide a way to track the therapeutic processes for real‐time molecular imaging, demonstrating high performance in studying the interaction of nanocarriers and MDR cancer cells/tissues in laboratory study and further shedding light on elaborate design of nanocarriers that could overcome MDR for clinical translation.

## 1 Introduction

This is an open access article under the terms of the Creative Commons Attribution License, which permits use, distribution and reproduction in any medium, provided the original work is properly cited.

Cancer is one of the leading threats to human health worldwide, accounting for tens of millions of deaths yearly and rapidly raising cancer incidence rates. Early diagnosis and efficient treatment of cancer are still challenges to be overcome. Besides, cancer multidrug resistance (MDR) heavily hampers the therapeutic efficiency and leads to high recurrence rate and therapeutic failure.^1^ Increasing evidences demonstrate that MDR is one important factor to cause recurrence and refractory of cancer, resulting in reduced survival in several tumor types.^2^ In general, MDR cancer cells exhibit cross‐resistance to a broad range of chemotherapeutic agents. Although many mechanisms are investigated to be involved in cancer MDR, further study is still required.^3^ Therefore, clarifying the mechanisms in MDR cancers and finding effective strategies to overcome MDR for chemotherapy are crucially important to validate and promote cancer therapeutic outcomes.

In recent decades, the rapid development of nanomedicines has attracted much attention for cancer therapy,^4^ because the nanoscale vehicles, which mainly refer to those with diameters <200 nm, can selectively accumulate in solid tumors through enhanced permeability and retention (EPR) effect.^5^ Besides, the nanocarriers can also be decorated with various kinds of targeting moieties to specifically interact with cancer cells.^6^ As a result, the nanocarriers have the capability to effectively deliver therapeutic payloads to tumor tissues to maximize the therapeutic effects, while minimizing the side effects, such as systemic toxicity. Moreover, most of the imaging‐functionalized inorganic nanocarriers could be utilized as probes to track their location, investigate drug release, and monitor the therapeutic efficacy.^7^, ^8^ In recent years, nanocarriers have demonstrated a high potential to overcome cancer MDR for therapy.^9^, ^10^, ^11^ This Review summarizes the biological mechanisms involved in cancer MDR and recent advances in applying inorganic nanocarriers to overcome MDR for cancer theranostic.

## 2 Mechanisms of Cancer Multidrug Resistance

### 2.1 Cellular Drug Resistant Mechanisms

Cancer cells are the main undertakers and actors of drug resistance. The cellular drug resistant mechanisms can be classified into two major categories: classical efflux transporters‐based mechanism and nonclassical resistance mechanism.

#### 2.1.1 Classical Efflux Transporter‐Based Mechanism

The most encountered drug resistance is caused by increased drug efflux from cancer cells, which is mediated by the ATP binding cassette (ABC) family of membrane transporters.^12^ Until now, over 48 types of ABC transporters have been identified in humans, and over 12 of them have been reported to cause drug resistance. The structure of ABC family transporters is generally formed by two transmembrane regions. The two transmembrane regions usually contain several transmembrane domains and two ATP binding cassettes in the cytoplasm, while the ATP binding cassettes play an essential role in cleaving ATP to generate energy for nutrients and small peptides across membranes. Among the transporters, the P‐glycoprotein (P‐gp, also known as ABCB1 or MDR1), a 170 kDa MDR1 gene product, is the most extensively studied and characterized ABC transporter.^13^ The expression of P‐gp has been identified in a wide range of MDR cancers. P‐gp has a large polymorphous drug‐binding domain within the transmembrane segments, which could be responsible for drug recognition.^14^, ^15^ It can recognize and bind to a wide range of electrically neutral or positively charged hydrophobic drugs including many conventional anticancer drugs, such as anthracyclines (e.g., doxorubicin (Dox) and daunorubicin (DNR)), vinca alkaloids (e.g., vincristine and vinblastine), podophyllotoxins (e.g., etoposide), and taxanes (e.g., taxol), and stimulate its ATPase activity, resulting in shape transformation of P‐gp and drug release into either the outer leaflet of the membrane or extracellular space, which finally reduces the drug efficacy.

However, it was also reported that some MDR cancers did not express P‐gp but still exhibited the MDR phenomenon,^16^ and then other efflux pumps were found. For instance, Deeley and co‐workers identified another ABC family of MDR‐associated protein 1 (MRP1) in an MDR lung cancer cell line.^17^ The MRP1 has a similar structure to P‐gp and only lacks the five‐transmembrane domains at the amino‐terminal of the P‐gp core; but, unlike P‐gp, the MRP1 could recognize negatively charged drugs and drugs with glutathione conjugation, glucosylation, sulfation, and glucuronylation.^18^ The discovery of MRP1 indicates the finding of other homologs of ABC family transporters such as the other eight ABCC subfamilies, six of which have been confirmed to be involved in the exclusion of anticancer agents and antiviral compounds.^19^, ^20^ Besides P‐gp and MRP1, other MDR‐related membrane transporters (e.g., the breast cancer resistance protein BCRP, also known as ABCG2) are also important ABC transporter,^21^, ^22^, ^23^ which plays an essential role in resistance to a variety of cancer therapeutic agents, including mitoxantrone, topotecan, doxorubicin, and SN‐38.^24^ Moreover, other ABC family transporters have also been found to be associated with drug resistance, such as the “sister of P‐gp,” ABCB11, and ABCA2, although their roles in cancer multidrug resistance remain unclear.^25^, ^26^

#### 2.1.2 Nonclassical Mechanism

The nonclassical mechanisms refer to nontransporter based MDR mechanisms, which provide drug resistance of cancer cells by mainly relying on reduced drug uptake instead of the efflux transporters‐based MDR procedures. The hydrophilic drugs usually can enter into cells by piggybacking on transporters that are responsible for transporting nutrients and specific agents into cells through endocytosis, but it may fail to accumulate inside MDR cancer cells instead of the efflux effect.^27^ For instance, Shen et al. found that cisplatin‐resistant cancer cells showed pleiotropic defects, associated with reduced plasma membrane protein, which finally reduced accumulation of drugs and became cross‐resistance to anticancer drugs,^28^ while there was no obvious overexpression of energy‐dependent efflux transporters. Besides this, the activation of the detoxifying system also could cause MDR cancer cells; for instance, the glutathione S‐transferase (GST) is a family of enzymes involved in drug and xenobiotic detoxification. Generally, GST is ubiquitously expressed in most living organisms to protect cells from the attack of reactive electrophiles, as it could catalyze the biotransformation processes and productions of execrable polar molecules by reacting with glutathione (GSH).^29^ Several drug resistant cancer cell lines have exhibited the overexpression of GST. In addition, coordinated induction of the MDR transporter P‐gp and the detoxifying enzyme were observed in drug‐resistant cancer cells; for instance, the P‐gp and GST‐π isoenzyme were co‐overexpressed in MCF/ADR cells, leading to an increased peroxidase activity.^30^ In addition to GST, glutathione (GSH) also plays a major role in MDR cancer cells by reacting with exogenous substrates and then removing them from milieu through ABC transporters, as elevated GSH levels have been investigated in many MDR cancer cells.^31^, ^32^

Furthermore, the defect of apoptotic pathway may also induce drug resistance, because the apoptotic threshold has been usually elevated in MDR cancer cells compared to that without drug resistant performance.^33^ Ceramide, an endogenous constituent of the lipid bilayer, plays a critical role in various signal pathways, including immune response and apoptosis, which are related to MDR.^34^ Generally, ceramide activates the apoptotic pathway when cancer cells encounter cellular insults, such as radiation or chemotherapeutic agents.^10^, ^35^ However, the glucosylceramide synthase could decrease the ceramide level in MDR cancer cells by inducing inactivation of ceramide to subsequently increase the apoptotic threshold, making cancer cells less sensitive to therapy.^36^ Another potential factor to elevate the apoptotic threshold is the Warburg effect, by which the cancer cells produce energy mainly by glycolysis.^37^ However, excessive glycolysis in cancer cells renders the outer mitochondrial membrane (OMM) less susceptible to permeabilization, which leads to elevated apoptotic threshold^38^, ^39^ because the apoptotic pathway requires regular permeabilization of OMM to release the mitochondrial proapoptotic proteins. Thus, the extent of extra or intracellular insults, which are able to induce apoptosis in normal cancer cells, is ineffective to MDR cancer cells.

### 2.2 Extracellular Drug Resistant Mechanisms: Vasculature and Hypoxia

Some types of cancer cells are sensitive to chemotherapeutic agents in monolayer cultures but become less sensitive to the same type of tumor in animals, indicating the tumor microenvironment is related to drug resistance. The pathological characteristics of tumor microenvironment including abnormal vasculature, hypoxia, acidic pH, increased interstitial hyper‐tension, and lack of lymphatics could affect the response of tumor cells to anticancer drugs, tumor growth, and multidrug resistance.^40^ The highly disorganized and constantly changing tumor vasculature is regarded as one of the most striking and key factors in tumor phenotype evolution.^41^ For example, the vessel in tumors is often dilated, torturous, and highly disorganized spatially, associating with angiogenesis and vascular destruction, which all together rises to form a “core–shell” structure in tumors with a hypoxic core and proliferating outer shell.^42^ The interstitial hypertension and “core–shell” structure make it difficult to supply nutrients and oxygen to the center of tumors, and it also limits the penetration of anticancer drugs to access tumor cells there, finally causing drug resistant to chemotherapy. The drug resistant related tumor hypoxia could be detected by MRI with pH‐responsive nanocarriers, as it could release more contrast agents (CAs; i.e., Mn^2+^) at lower pH to argue higher contrast enhancement.^43^ Because the excessive consumption of glucose and over production of lactate in tumor hypoxia regions lead to a lower pH in the hypoxia locations compared to that in surrounding tumor tissues.

The remarkable consequence of the aberrant tumor vasculature is the decreased oxygen levels in hypoxic regions, which is another potential factor to trigger cancer drug resistance. Hypoxia is generally caused by excessive oxygen consumption and insufficient oxygen/glucose supply, and hypoxic cancer cells have been recognized as more resistant to chemotherapy.^44^ Inadequate oxygen supply could induce cancer cells to initiate a complex phenotype transformation such as hypoxia inducible factor‐1 (HIF‐1), which acts as the master regulator of hypoxia.^45^ The HIF‐1 is a heterodimer comprised of HIF‐1α and HIF‐1β subunits, and it is regulated by oxygen concentrations. In hypoxia, HIF‐1α could enter nucleus from cytoplasm to form active heterodimer with a beta subunit of HIF‐1β, while during this process, it binds to the hypoxia responsive elements and induces the expression of hundreds of target genes. These genes are extensively involved in drug efflux, apoptosis, DNA damage repair, autophagy, and others; for instance, a vast array of HIF‐1 downstream genes was found to be associated with MDR.^46^, ^47^, ^48^

### 2.3 Cancer Stem Cells and Drug Resistance

Tumors are recognized as heterogeneous with hierarchies of cellular populations, and a minority population of cancer cells acts as cancer stem cells (CSCs, also called tumor initiating cells), which possess the capacity of long‐term renewal and are responsible for tumor initiation, growth, and recurrence.^2^ Until now, CSCs have been reported in several types of solid tumors, including colorectal, breast, pancreatic, lung, and hepatocellular.^49^, ^50^, ^51^, ^52^ More and more in vivo evidence demonstrated that CSCs could promote MDR. Generally, CSCs are regarded as quiescent and nondiving cells, resist conventional treatment of radiotherapy or chemotherapy, and finally rise to recurrence and tumor relapse. Even worse, the recurrent tumors often produce a population of MDR cancer cells, in order to make it more malignant, that spread quickly and are resistant to radiotherapy or previously treated drugs. CSCs have been reported to express a high level of drug efflux proteins, such as P‐gp, MRP1, and BCRP, to help CSCs avoid damages from cytotoxic drugs and resist to chemotherapy.^2^, ^27^, ^53^ Furthermore, CSCs also demonstrated active DNA‐repair and detoxification capacity, for example, the expression of Aldehyde dehydrogenases1 (ALDH1), a molecular metabolic detoxification enzyme for catalyzing oxidation of aldehyde formation in alcohol metabolism,^54^ has been frequently elevated as investigated in some CSC cell lines to activate tumor recurrence.^55^ The ALDH1 has implications in drug detoxification and chemo‐resistance associated with poor therapeutic outcomes.

## 3 Inorganic Nanocarriers Overcoming Drug Resistance for Cancer Theranostic

**Figure 1:**
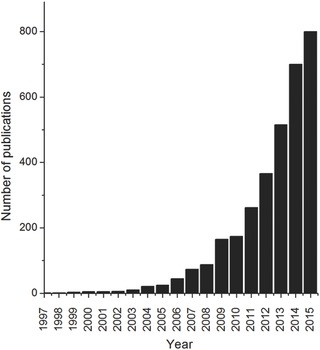
The statistics of published scientific papers related to “nanoparticles” and “multidrug resistance” from 1997 to 2015 (data obtained from Web of Science, until December 31, 2015).

**Figure 2:**
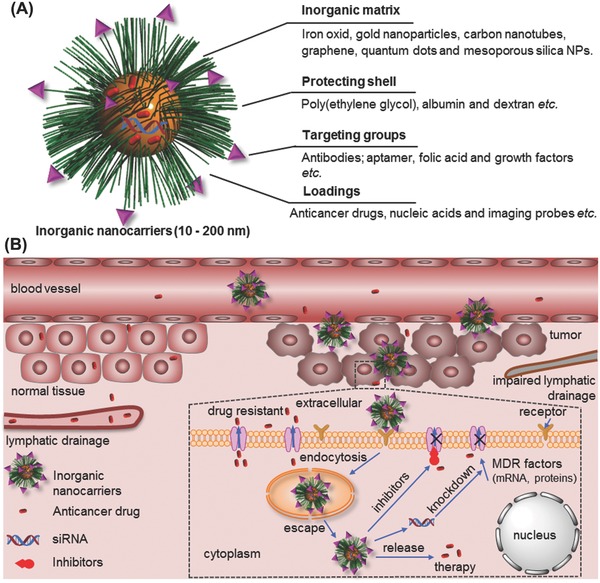
A) Schematic representation of multifunctional inorganic nanoparticles carrying multiply therapeutic agents (drug, inhibitor, siRNA). B) Schematic representation of multifunctional inorganic nanoparticles as the platform to reverse the MDR in resistant cancer cells.

Conventional drugs are difficult to effectively treat cancers, especially MDR cancers, as there are several critical obstacles, such as limited drug solubility, poor pharmacokinetics/bio‐distributions, lacking effective tumor selectivity, and failure to penetrate through the highly dense extracellular matrix.^56^ Nanocarriers are promising platforms to overcome MDR with their unique physical properties and biological performances.^4^, ^57^, ^58^ Recently, the research of employing nanoparticles for reversing MDR has increased dramatically in the global scale (**Figure** **1**). Unlike conventional drugs that diffuse systemically without targeting tropism, nanocarriers can selectively accumulate in tumor tissues through passive/active targeting processes (**Figure** **2**B), and numerous nanocarriers have been developed for drug delivery, such as lipids nanoparticles, polymer conjugates, liposomes, dendrimers, micelles, and inorganic nanocarriers. The inorganic nanocarriers (e.g., iron oxide nanoparticles,^59^ quantum dots (QDs),^60^ gold nanoparticles,^61^ carbon‐based nanocarriers,^62^, ^63^ and silica nanoparticles^64^) have exhibited distinctive advantages for drug delivery, including high surface‐to‐volume ratio, controllable size and shape, potential imaging function, tunable pore structures and facile surface modification.

The inorganic nanocarriers could be featured with stimuli‐responsive release function, which could specifically release drugs in tumor microenvironments when responding to external triggers (e.g., hyperthermia, light, and magnetic field) to avoid drug efflux transporters recognizing anticancer drugs; this represents the “Trojan horse” style of drug delivery.^65^ Meanwhile, anticancer agents as well as therapeutic genes (e.g., DNA or siRNA) could be loaded in inorganic nanocarriers to achieve synergistic therapeutic effects due to their ease of surface modification (Figure 2A).^66^ Moreover, inorganic nanocarriers could provide multifunctional platforms for cancer treatment, such as hyperthermia therapy,^67^, ^68^ which demonstrated potential to reverse MDR in cancers by combination with chemotherapy.^69^ More importantly, these inorganic nanocarriers could also provide molecular imaging function, which could help monitoring drug delivery processes and therapeutic outcomes, leading to improved treatment efficacy, especially for treating MDR cancers.^70^, ^71^

### 3.1 Iron Oxide Nanoparticles

**Figure 3:**
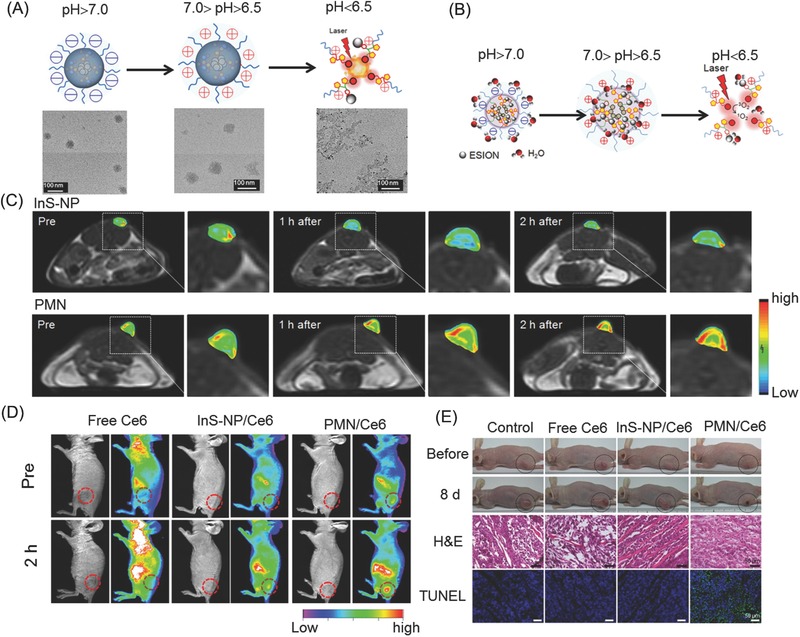
A) pH‐dependent structural transformation behavior in PMNs. B) Schematic representation of pH‐dependent structural transformation in PMNs. C) In vivo T1‐weighted MR images and color‐mapped images of tumor sites before and 1 or 2 h after intravenous injection of PMNs or InS‐NPs into HCT116 tumor‐bearing nude mice. D) In vivo NIR imaging of HCT116 tumor‐bearing nude mice after intravenous injection of PMNs, InS‐NPs, or free Ce6. E) H&E and TUNEL staining of tumor tissue sections to determine treatment effectiveness in terms of tumor cell death by apoptosis. Adapted with permission.^77^ Copyright 2016, American Chemical Society.

With excellent biocompatibility and unique physical/surface properties, iron oxide nanoparticles have been extensively studied for a diverse array of biomedical applications, such as CAs for MRI, hyperthermia treatment, drug delivery, bio‐sensing, and protein separation.^72^ In particular, the superparamagnetic iron oxide nanoparticles (SPIOs) could shorten the T2 relaxation time in tissues, and ten types of SPIOs have been approved as T2 type of CAs for clinical MRI.^73^ Besides, iron oxide nanoparticles can increase the internalization between drugs and cancer cells, delivering bioactive agents into cytoplasm through the endocytosis pathway, while escaping the recognition by pump transporters on the surface of MDR cancer cells. For instance, by binding Dox to polyethylenimine (PEI) through pH sensitive linkers, the Dox‐PEI was then loaded on the surface of iron oxide nanoparticles. The Dox could escape from the recognition of some ABC transporters of glioma cells, because the Dox loaded nanoparticles can achieve endosomal escape by the “proton sponge effect” of PEI to disrupt endosomes, and finally reach the cytoplasm to achieve higher intracellular accumulation of drugs in Rat glioma C6 drug‐resistant cells (C6‐ADR) compared to that of free Dox.^74^ In another study, the Dox‐conjugated iron oxide nanoparticles through labile bonds demonstrated the ability to traverse in intracellular milieu and release Dox in endosomes to make it less susceptible to P‐gp mediation.^75^ By combination with anti‐ABCG2 monoclonal antibodies (mAbs), anticancer drugs‐loaded iron oxide nanoparticles can significantly inhibit the proliferation and migration of CSCs, and further lead to an obvious suppression of tumor growth in CSC‐transplanted mice.^76^ Recently, Ling et al. developed pH‐sensitive magnetic nanoparticles (PMNs), which can disassemble in the acidic tumor microenvironment (**Figure** **3**A,B) and switch the surface charge of nanoparticles; this resulted in an increase in cell adsorption and permeation.^77^ In addition, the disassembly would bring pH‐responsive T1 MR contrast and fluorescence, which enabled early stage diagnosis of tumors (Figure 3C,D). Furthermore, the pH‐sensitive nanoparticles (PMNs) demonstrated pH‐triggered generation of singlet oxygen and the ability to kill resistant cancer cells compared to nonsensitive ones (InS‐NPs) when employed in loading photosensitizers for photodynamic therapy (Figure 3E).

Modulators are frequently investigated to restore the MDR property, which demonstrates the potential to affect MDR transporters. However, their unfavorable pharmacokinetics and side effects make it difficult to overcome drug resistance directly, even when combined with chemotherapeutics.^27^ The difficulty may be due to the differences of pharmacokinetics and tumor accumulation between the anticancer drugs and modulators, making it difficult to achieve synergistic effects.^78^ Interestingly, nanocarriers that could co‐deliver both inhibitors and anticancer drugs to cancer cells would overcome this drawback and achieve synergistic effects. Recently, it was reported that the anticancer drug DNR and potential MDR modulator 5‐bromotetrandrine (5‐BrTet) co‐loaded with iron oxide nanoparticles could increase the accumulation of loaded compounds (5‐BrTet and DNR) and downregulate the expression of mdr‐1 gene, ultimately resulting in significant therapeutic effects in MDR cancer cells.^79^

Another strategy is to downregulate the expression of MDR‐related proteins by RNA interference, as siRNAs could help to treat drug resistance by reducing the protein levels of P‐gp, MRP1, and Bcl2.^80^, ^81^ However, directly administrating siRNA faces the obstacles of degradation and elimination by ribonuclease (RNase), poor permeability, and endosomal trapping, while nanocarriers could overcome those barriers for delivering siRNA to MDR cancer cells. By modifying the surface of iron oxide nanoparticles, it could efficiently carry MDR protein‐silencing siRNA by electrostatic interaction, protect them from the attack of RNase, and further enhance the internalization of loaded siRNA with cancer cells as well as help them escape from endosome. For example, polycations wrapped iron oxide nanoclusters can efficiently incorporate P‐gp regulation siRNA to silence the target messenger RNA, resulting in a significant reduction of P‐gp expression and ultimately restored drug sensitivity of MDR cancer cells, while the iron oxide nanoclusters part could provide significantly T2 contrast for MR imaging to monitor the location of nanocarriers as well as the therapeutic effects.^80^

Besides using siRNAs, hyperthermia also demonstrated the potential to reverse MDR cancers associated with chemotherapy. Hyperthermia is a fairly new approach for cancer treatment that is produced by magnetic nanoparticles in magnetic field that interact with each other to generate heat at tumor sites to change the physiology of cancer cells to induce apoptosis.^67^, ^82^ Hyperthermia could also trigger drug release to enhance drug delivery to MDR tumors. By co‐delivering chemotherapeutic drugs and inhibitors, the iron oxide nanoparticles in the alternating magnetic field can decrease the expression of MDR‐related proteins to subsequently inhibit tumor growth.^83^ Furthermore, it was found that iron oxide nanoparticles can effectively eliminate CSCs through magnetic hyperthermia to relieve drug resistance of tumors by a series of effects, such as acute necrosis and generation of reactive oxygen species.^84^

Overall, iron oxide nanoparticles can load anticancer drugs, nucleic acids, and chemical inhibitors to achieve synergistic effects for cancer therapy, including more effective treatment of MDR cancers compared to the single functional treatment modalities. Besides, magnetic hyperthermia has the potential to reverse MDR cancer cells. Moreover, it provides a “theranostic” platform with therapy and diagnostic functions to monitor the therapeutic efficacy besides treatment. Regarding those progresses, several critical issues still remain for applying it in MDR tumor treatment. One issue is that the magnetic properties of iron oxide nanoparticles (e.g., magnetic susceptibility) still require modification with higher sensitivity for cancer detection by MRI.^85^, ^86^ The development of T1–T2 dual‐modal contrast agents will potentially solve this drawback, as it can simultaneously provide T1‐weighted imaging with a high tissue resolution and T2‐weighted imaging with a high feasibility, for tumor detection.^70^, ^87^ Besides, the therapeutic agents are usually loaded on the surface of iron oxide nanoparticles, which is difficult to carry and deliver sufficient dose of drugs to MDR cancers.^88^ The iron oxide nanoparticles with hollow porous structures may load more drug molecules for MDR tumor treatment.

### 3.2 Quantum Dots

QDs are a new class of fluorescent probes for molecular imaging with unique photophysical properties, such as size‐tunable light emission, high signal brightness, simultaneous excitation of multiple fluorescence colors, and resistance against photobleaching.^89^ QDs are another versatile type of nanocarriers that contain dual‐imaging and therapeutic functions to overcome drug resistance of cancers.

**Figure 4:**
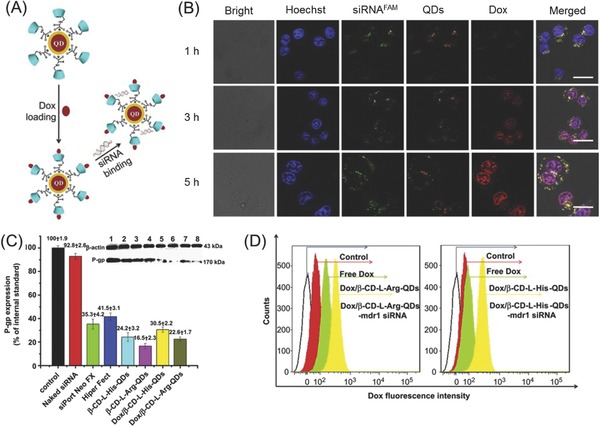
A) Schematic illustration of siRNA and Dox co‐loading onto L‐amino acid‐b‐CD‐modified QDs. B) Confocal microscopy images of siRNA^FAM^ binding and Dox loading in QDs into HeLa/Dox cells. C) The knockdown of P‐gp through Pgp‐siRNA loaded QDs in resistant cancer cells. D) Quantification of the amount of Dox in HeLa/Dox cells by flow cytometry. Adapted with permission.^91^ Copyright 2016, Elsevier.

Like iron oxide nanoparticles, QDs can also be engineered as drug delivery platforms for delivering siRNAs and anticancer drugs by the surface modification.^90^ For instance, Li et al. developed L‐Arg or L‐His modified beta‐cyclodextrin (β‐CD) coated with CdSe/ZnSe QDs to simultaneously deliver DOX and P‐gp targeted siRNA to reverse the MDR HeLa cancer cells.^91^ Dox can be loaded into the hydrophobic parts of β‐CD, while the P‐gp related siRNA was absorbed onto the surface of QDs by electrostatic interaction between the positive charging L‐Arg/L‐His with the negative charging siRNA (**Figure** **4**A). The multifunctional QDs could help the drugs bypass P‐gp‐mediated drug efflux and reduce the mRNA levels with lower mdr1 and P‐gp expression (Figure 4C). The intracellular accumulation of Dox delivered by QDs was two to threefold higher compared to that of free drugs. In addition, the accumulation of drugs increased five to sixfold when further co‐delivered with P‐gp targeted siRNA (Figure 4D). Finally, the Dox and siRNA co‐encapsulated QDs could efficiently induce apoptosis of cancer cells, while the imaging function could trace the nanocarriers inside cancer cells by laser confocal microscopy in a real‐time manner (Figure 4B).

In the past two decades, QDs have been widely exploited for biomedical applications including diagnosis and drug delivery due to their unique optical and chemical properties.^92^ However, the safety consideration of QDs becomes one of the most critical issues beyond their clinical translation, which was mainly caused by the release of free heavy metal ions from QDs and the production of reactive oxygen species (ROS) that can damage cellular proteins, lipids, and DNA.^93^ Besides, the physicochemical and surface characteristics, such as shape, size, and surface charge as well as coverage, also play essential roles in the toxicity performance of QDs. In addition, long‐term toxicity of QDs is also another consideration.

### 3.3 Gold Nanoparticles

Gold nanoparticles have been intensively developed as effective nanocarriers for cancer therapy due to their biocompatibility, high tissue permeability, colloidal stability, and unique size properties.^94^ The surface of gold nanoparticles could be easily modified with several high affinity functional groups to carry bioactive compounds or for PEGylation. Besides, gold nanoparticles have been widely applied for imaging, such as for CT imaging owing with their high atomic number and electron density, for optical imaging with high optimal light scattering performances,^95^ for photoacoustic (PA) imaging with the produced thermal after laser irradiation,^96^ and for Raman spectroscopy (SERS) imaging with the enhanced scattering on their surface.^97^

Generally, bioactive compounds can be easily loaded onto gold nanoparticles through several different approaches, such as physical adsorption, ionic bonding, and covalent bonding. Gold nanoparticles can deliver drugs to intracellular spaces to highly enhance the efficacy against tumor cells,^98^ for instance, high efficacy of PEGylated gold nanorods (PEG‐GNRs) on reversing MDR cancer cells has been identified.^99^ For another example, the platinum (IV) prodrugs conjugated GNRs can evade the deactivation of overexpressed detoxification proteins including metallothionein and GSH in A549R cells, and for restoration of the drug sensitivity in cisplatin‐resistant cancer cells. Besides, the efficacy of anticancer agents delivered by gold nanoparticles can overcome MDR, which has been confirmed on several different types of MDR cancer cell lines.^100^, ^101^ In addition, the targeting ligands, polymers, and nuclei acids can be fixed onto the surface of gold nanoparticles to achieve higher accumulation in tumor tissues for enhancing the therapeutic efficacy. Gold nanoparticles with antiandrogen ligands have been developed to target MDR prostate cancer cells with high efficacy of drugs,^102^ which can selectively target two kinds of receptors including rogen receptors and G‐protein coupled receptors (GPRC6A), while both receptors were usually upregulated in MDR prostate cancer cells. In addition, gold nanoparticles can be developed as co‐delivery systems for drug and MDR sensitize agents (modulators or siRNAs) to restore the drug sensitivity of MDR cancer cells. For example, Rajput et al. formulated octadecylamine‐modified gold niosomes, in which inner hydrophobic core can be used to load hydrophobic drugs (thymoquinone), while the outer layer of octadecylamine can bind and carry negatively charged siRNA through electrostatic interaction.^103^ The drug and siRNA co‐loaded niosomes could restore the MDR properties by reducing the expression of Akt to induce enhanced apoptosis of cancer cells both in vitro and in vivo tests.

**Figure 5:**
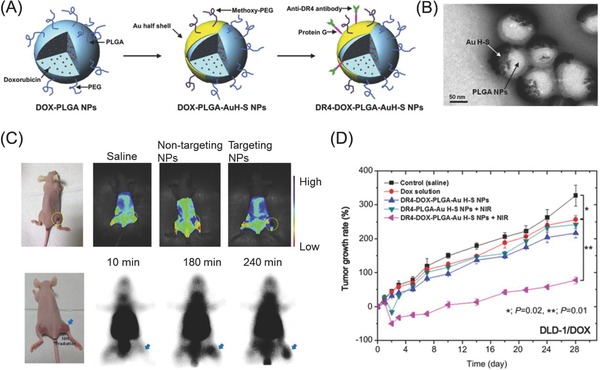
A) Schematic diagrams showing the construction of DR4‐DOX‐PLGA‐Au H‐S NPs. B) TEM image of DR4‐DOX‐PLGA‐Au H‐S NPs. C) Upper: In vivo NIR absorbance images of DLD‐1/DOX tumor‐bearing mice measured post 24 h injection of DR4‐PLGA‐Au H‐S NP solution. Down: 99mTc‐MIBI scintigraphy images at different time point after NP injection on a DLD‐1/DOX tumor‐bearing mouse. D) Relative tumor volume change in DLD‐1/DOX tumor‐bearing mice. Adapted with permission.^108^ Copyright 2016, Elsevier.

In addition to carry bioactive compounds, gold nanoparticles can be applied for cancer photothermal therapy (PTT), as it can efficiently convert absorbed light to heat to destroy adjacent cancer cells without the destruction of surrounding normal and healthy tissues.^104^ By changing the thickness of the core and shell of gold nanostructures, the peak of localized surface plasma resonance can be shifted to the near‐infrared radiation (NIR) region, which is beneficial for tumor treatment, as NIR light demonstrates deeper light penetration in tissues than the light with wavelength below 700 nm. There are numerous studies applying gold nanoparticles for cancer PTT in preclinical and clinical trials.^105^, ^106^ It was reported that anti‐HER^2+^ coated silica/gold nanoshells could selectively bind to MDR HER^2+^ breast cancer cells to overcome drug resistance and kill cancer cells with PTT.^107^ Moreover, by combining PTT with chemotherapy, it could induce synergistic effects and eliminate MDR cancer cells more effectively. For instance, the DOX‐loaded‐poly(lactic‐co‐glycolic acid) hybrid gold nanoparticles functionalized with targeting moieties of anti‐death receptor‐4 monoclonal antibody (**Figure** **5**A,B) were employed to treat MDR cancers by chemo‐photothermal therapy.^108^ These nanoparticles could increase the amount of drugs delivered to tumors and decrease the activity of P‐gp by heat generated from Au nanoparticles after laser irradiation. The generated heat could offer effective therapy even with low dose of drugs and cause a large reduction in the growth rate of MDR tumor xenograft compared to free Dox (Figure 5C,D).

Overall, gold nanoparticles have been widely designated as multifunctional optical and CT probes for imaging, while peptides, polymers, proteins, drugs, oligosaccharides, and nucleic acids can be attached on the surface for cancer therapy. Besides, it could be used for tumor ablation by PTT, especially for treating MDR cancers by combining with other therapeutic methods. However, the synthetic method of some gold nanoparticles, such as nanoshells, nanostars, and nanorods, is quite complicated, while some stabilizing agents used for synthesis are toxic to normal cells.^109^ Moreover, some surface‐modified gold nanoparticles are less stable in saline solutions; thus, more biocompatible molecules, such as PEG, transferrin, and phospholipids, are required for surface modification to improve the stability of gold nanoparticles.^110^

### 3.4 Carbon‐Based Nanocarriers

Recently, carbon‐based materials have drawn much attention for biomedical applications due to their unique properties of high surface‐to‐volume ratio, thermal conductivity, rigid structural properties, and easy surface modification, which also demonstrate high potential for MDR cancer theranostics.

#### 3.4.1 Carbon Nanotubes

Due to their unique physical properties, carbon nanotubes (CNTs) have been widely investigated as a promising imaging probe, drug/gene delivery system, and thermal agents for cancer theranostics.^111^ Based on their structure, CNTs can be divided into two groups: multi‐walled carbon nanotubes (MWCNTs) and single‐walled carbon nanotubes (SWCNTs), both of which can be functionalized with pyrrolidine rings to attach a wide variety of biomolecules, imaging agents and drugs.^112^ Moreover, the unique physical properties of CNTs make them as promising imaging probes, because SWNTs can produce fluorescence in the NIR range for NIRF imaging, and as Raman probes can be used for biological imaging with their strong resonance Raman scattering and large scattering cross‐section.^113^ In addition, they can be exploited for PA imaging with strong absorbance in the NIR region and for MR imaging due to the contained impurities of metal nanoparticles, which render carbon nanotubes as promising multimodal imaging probes.

**Figure 6:**
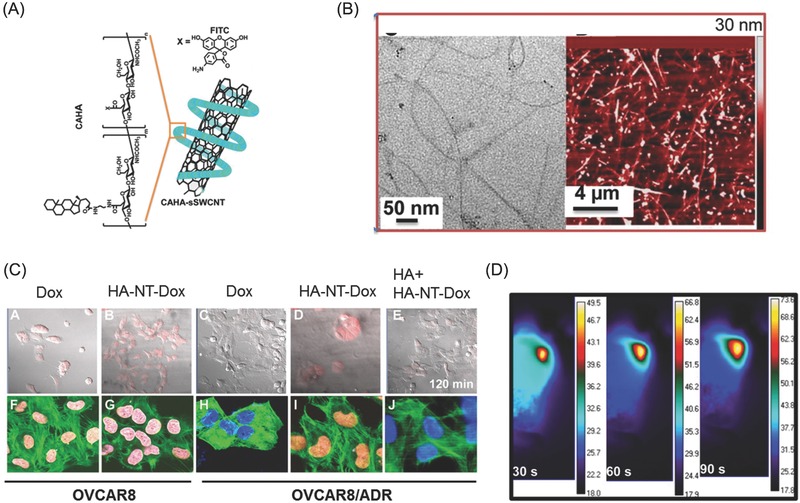
A) Schematic representation of cholanic acid‐derivatized hyaluronic acid wrapped semiconducting SWCNTs (CAHA‐sSWCNTs). B) TEM and AFM images of CAHA‐sSWCNTs. C) DOX uptake in DOX‐sensitive cancer cells (OVCAR8) and DOX‐resistant cancer cells (OVCAR8/ADR) cells. D) Thermal images of OVCAR8/ADR tumor bearing mice when exposed to 808 NIR laser 24 h post CAHA‐sSWCNT‐DOX injection. Adapted with permission.^117^ Copyright 2016, American Chemical Society.

The PEGylated SWCNTs can carry drugs and efficiently penetrate into mammalian cells without inducing obvious damage to plasma membrane, while avoiding the exclusion by MDR cancer cells, as it could accumulate and retain high level of drugs inside MDR cancer cells to overcome multidrug resistance.^114^ Recently, Wu et al. designed and synthesized CNTs with inner diameters of 40 nm through template synthesis, which can generate consequential resistive heat under magnetic field induction. These CNTs could deliver paclitaxel and C6‐ceramide to drug resistant pancreatic cancer cells to cause 71.5% of apoptosis in an on‐demand way upon the magnetic field induction, while no obvious toxicity to cancer cells was investigated without magnetic field induction, even when administrated with equal doses of paclitaxel‐loaded CNTs.^115^ Such a magnetic responsive delivery system may also be applied for delivering therapeutic nucleic acid (DNA or siRNA), antibodies, growth factors, and recombinant protein to overcome the MDR property of cancer cells. For instance, the Dox‐loaded, P‐gp antibodies (anti‐Pgp) functionalized SWNTs (Ap‐SWNTs) demonstrate high drug loading efficacy and NIR controlled drug release.^116^ The Ap‐SWNTs were specifically sensitive to MDR human leukemia cells (K562R) with 2.4‐fold higher cytotoxicity and caused significant cell death when against K562R with near‐infrared radiation compared to free Dox, because the P‐gp antibody functionalized SWNTs could effectively interact with P‐gp, which were ubiquitously overexpressed on the membrane surface of MDR cells. Recently, another study showed that the cholanic acid‐derivatized hyaluronic acid (CAHA) biopolymer wrapped SWCNT (diameter: 24 nm; core diameter: 0.7–0.9 nm; length distribution: 0.22 μm) (**Figure** **6**A,B) could effectively deliver chemotherapeutic agents to treat drug resistant OVCAR8/ADR cancer cells by targeting CD44. The CAHA‐sSWCNTs have a diameter of 24 nm and a length distribution of 0.22 μm, which is stable in serum medium and without obvious cytotoxicity. Furthermore, these drug‐loaded SWCNTs could affect the viscoelastic property to achieve high level of drug accumulation in tumors with strong cell killing effects (Figure 6C,D).^117^

#### 3.4.2 Graphene

Graphene is a 2D crystal graphite with sp^2^‐hybridized carbon atoms arranged in one‐atom thick structures, including few‐layer graphene, grapheme nanosheets, reduced graphene oxide (GO), and GO. Graphene and its derivatives are innate NIR fluorescence probes due to their intrinsic optical properties, while GOs are intrinsically good probes for photoacoustic imaging due to their strong absorbance in the NIR region. They can be labeled with external probes for multifunctional imaging, such as Cy7 for fluorescence imaging, radio nuclear for positron emission tomography imaging, and iron oxide nanoparticles for MR imaging.^111^, ^118^, ^119^ The planar structure of graphene also offers a high capacity to load other substances, such as drugs, polymers, and biomolecules. For instance, adriamycin can be loaded onto the surface of GOs with a high loading efficacy only by physical mixing.^120^ The adriamycin‐loaded GOs could escape the efflux action of P‐gp to achieve high accumulation of drugs inside cancer cells, resulting in much higher cytotoxicity against MDR MCF‐7/ADR cells compared to free adriamycin. Similarly, hematin‐terminated dextran (HDex) can be attached to the surface of GOs through π–π interaction to form graphene‐based nanohybrids, which can carry sufficient Dox to effectively kill MDR MCF‐7/ADR cells.^121^ As mentioned above, co‐delivery of MDR‐reversing agents and anticancer drugs is an effective approach to overcome MDR. For instance, siRNA has been co‐delivered with anticancer drugs by graphene for treating MDR cancers. The multifunctional composites of polyethylenimine/poly(sodium 4‐styrenesulfonates)/graphene oxide can simultaneously deliver adriamycin and miR‐21‐targeted siRNA to drug‐resistant cancer cells with high accumulation and finally enhance therapeutic efficacy by silencing drug‐resistance‐related miR‐21.^122^ In addition, with high absorption in the NIR region, the GOs could be applied for tumor ablation by PTT. Thus, the drug‐loaded GOs were dual functionalized for chemotherapy and PTT to achieve synergistic effects, demonstrating a promising strategy to treat MDR cancers.^69^, ^123^, ^124^

With their unique chemical and physical nature, the carbon‐based nanocarriers are promising platforms for cancer theranostic, including molecular imaging, PTT, targeted chemotherapy, and RNA interference. However, the pristine carbon‐based materials are usually insoluble in biological media and tend to aggregate with each other, so further modification with biocompatible materials on their surface and solutions to carry bioactive compounds is required. Besides, some noncovalent functionalization of bioactive compounds to graphene may not be sufficiently stable in physiological environments. Moreover, the properties of intrinsic NIR fluorescence and Raman scattering may be dramatically changed after covalently binding functional molecules/materials on the surface of graphene.^125^

### 3.5 Mesoporous Silica Nanoparticles

Mesoporous silica nanoparticles (MSNs) have attracted much attention for drug and gene delivery, owing to their high specific surface area, large pore volume, tunable pore structure, and well‐defined surface property for further modification.^126^ Besides drugs, by encapsulating MRI CAs, such as T_1_‐weighted MnO or T_2_‐weighted SPIO CAs, MSNs can be developed as mutilfunctional probes for MR cancer imaging,^127^, ^128^ while loading with NIR CAs or rare‐earth‐doped upconversion fluorescent nanoparticles, it also can be applied for optical imaging with high sensitivity.^129^

Until now, MSNs have been widely investigated to load anticancer drugs for overcoming MDR of cancer cells.^130^ Similar to other inorganic nanocarriers, the MSNs could efficiently incorporate drugs and effectively deliver them to tumor tissues through the EPR effect. The drug‐loaded MSNs could enter cancer cells and transport the drugs into cytoplasm to finally kill MDR cancer cells, as the MSNs could protect the drugs inside their core to avoid the recognition by the MDR‐related efflux transporters, while the free drugs could be recognized by those drug efflux transporters to be pumped out of cancer cells, leading to therapeutic failure. Specifically, the Dox‐conjugated MSNs through endosomal pH‐cleavable bonds could deliver Dox into cellular plasma by endocytosis, while avoiding the recognition of efflux pump of resistant cancer cells, resulting in enhanced therapeutic efficacy with increased cellular apoptosis.^131^ Besides, it was recently reported that the Dox‐loaded MSNs exhibited pore‐size‐dependent and sustained drug release performance, while larger pore size resulted in faster drug release and higher intracellular drug levels to cause strong MDR‐reversing effects.^132^ This was due to the effective cellular uptake, pH‐responsive drug release, and down regulation of P‐gp as well as ATP depletion. In another study, the reverse of MDR by MSN was also achieved by reducing the P‐gp expression and effective cell internalization.^133^ Furthermore, by modifying with TAT peptides on the surface of MSNs, they can deliver DOX directly into the nucleus and induce apoptosis of MDR MCF‐7/ADR cancer cells.^134^ Additionally, by co‐loading two anticancer drugs into MSNs could ultimately reverse the MDR in resistant cancer cells. For instance, the therapeutic effect of Dox‐loaded MSNs was enhanced by adding another anticancer drug of CPT, which decreased the tolerance of cancer cells to one type of anti‐cancer drug.^135^ The mechanism of applying drug‐loaded MSNs for overcoming cancer MDR was also studied.^136^, ^137^ Interestingly, Li et al. systematically analyzed the gene expression of MCF‐7/ADR cancer cells after treating with a nuclear targeting, Dox‐loaded MSNs (DOX@NT‐MSNs). It revealed that the DOX@NT‐MSNs could affect the apoptosis‐ and MDR‐related gene expression, and inactivate the DNA repair processes and disrupt the p53 pathway, all of which contributed together to the reversal of MDR.

**Figure 7:**
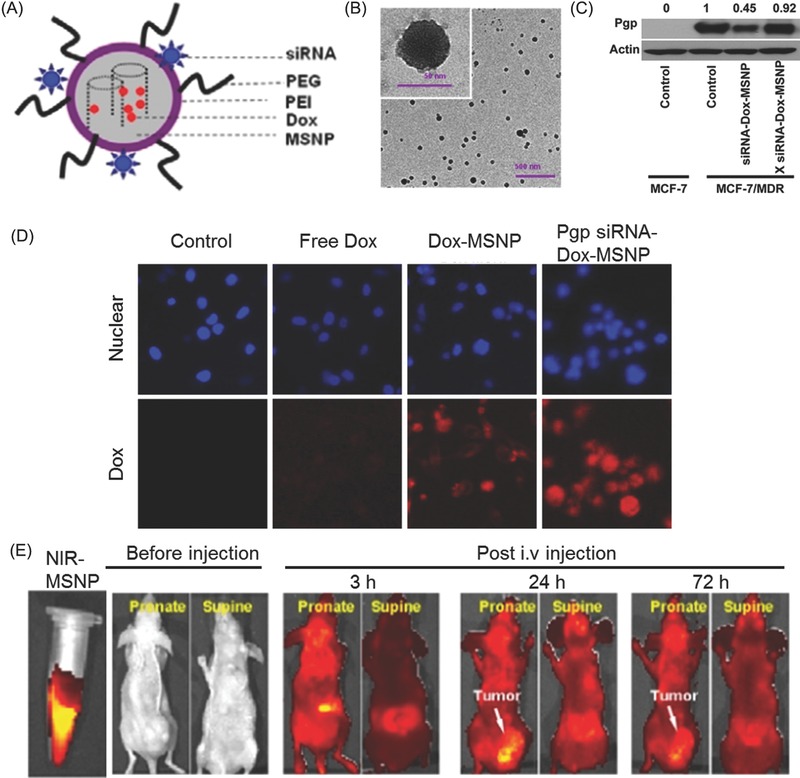
A,B) The scheme depicts MSNP coated PEI‐PEG co‐loading drug and nucleic acid and TEM imaging of these MSNP. C) Immunoblotting was carried out to measure the P‐gp expression after treatment of P‐gp‐siRNA loaded MSNP. D) Representative fluorescent images of the cells treated with Dox‐MSNP and Pgp‐siRNA‐Dox‐MSNP and free drug. E) Biodistribution of MSNP in an MCF‐7/MDR tumor model bearing nude mice after i.v. injection. Adapted with permission.^140^ Copyright 2016, American Chemical Society.

Besides anticancer drugs, MSNs can also deliver other types of MDR‐reversing agents, such as nucleic acids and chemosensitizers, achieving synergistic effects in treating MDR cancers. For instance, the MSNs could deliver cetyltrimethyl ammonium bromide (CTAB), a chemosensitizer for overcoming MDR and an anticancer drug of Dox to MDR cancer cells through pH‐triggered drug release. It finally led to effective MDR reversing function by synergistic cell cycle arrest and an apoptosis‐inducing effect.^138^ Besides, through surface modification, MSNs demonstrate the ability to effectively load nucleic acids and specifically deliver them to tumors.^139^ So far, MSNs have been investigated to carry several kinds of MDR‐related gene targeting siRNAs together with anticancer agents to restore MDR of cancer cells, such as MSNs‐based nanosystems loading with P‐gp targeting siRNA and Dox to overcome MDR MCF‐7/MDR cancer cells. Recently, Meng et al. reported a PEI‐PEG coated MSNs with size of 50 nm (**Figure** **7**A,B).^140^ The MSNs could effectively carry P‐gp‐related siRNA to downregulate the expression of P‐gp (Figure 7 C), leading to an enhanced accumulation of Dox in resistant cancer cells (Figure 7 D). The biodistribution of MSNs was monitored by assessing the fluorescence intensity in mice after labeling MSN with NIR dye (Figure 7 E). It revealed that 8% of MSNs was accumulated in tumor regions. The dual therapeutic agents in the MSNs could induce synergistic effects in inhibiting the growth of MDR cancer *in vivo*.

Overall, multifunctional MSNs have been developed to treat MDR cancers, and as CAs for tumor molecular imaging. However, several obstacles still remain for clinical applications. Although multifunctional MSNs demonstrate high performance for cancer theranostics, the synthetic approaches are limited and are still complicated for large‐scale production.^141^ Moreover, the biodegradation rate of MSNs still remains very low, which may raise toxicity concerns. Although the hybrid organic components in Si‐O‐Si frameworks could accelerate the biodegradation, the degradation still remains an important issue for inert Si‐O‐Si frameworks.^142^

## 4 Conclusion and Perspective

Cancer MDR has become a major impediment beyond successful cancer chemotherapy for decades, and overcoming cancer MDR is an important task for cancer treatment. In most cases, cancer drug resistance involves the mechanisms of decreased influx/increased efflux of drugs mediated by membrane‐based pump transporters, increased cell apoptosis thresholds, and tumor microenvironments. Strategies for the reversal of MDR are alteration of transporters that are responsible for drug efflux, modulation of proteins that are regulating apoptosis, and improving the uptake of drugs using nanocarriers. One common strategy is directly delivering drugs with inorganic nanocarriers to enhance the cellular uptake of drugs and to help them to escape the pump effects of transporters (e.g., P‐gp). Besides, by modifying inorganic nanoparticles with antibodies, peptides, and aptamer, it can target cancer cells with enhanced drug accumulation and high cancer cell selectivity. Another common strategy is to co‐load MDR‐reversing agents (e.g., nucleic acids and chemical inhibitors) together with anticancer agents in inorganic nanocarriers to achieve synergistic therapeutic effects on restoring the MDR cancer cells and promote the therapeutic efficacy. The MDR‐reversing agents, such as nuclei acids and inhibitors, could decrease the expression of MDR‐related proteins, such as Pgp, MRP proteins; inhibit the drug efflux pump; and enhance the expression of apoptosis‐related genes (e.g., p53 and TNF) to normalize the cell function, to finally decrease the biological sensitivity of MDR cancer cells to drugs. Furthermore, inorganic nanocarriers provide alternative approaches for cancer treatment besides chemotherapy (e.g., hyperthermia therapy) to effectively surmount MDR cancers. The synergistic therapy could increase the intracellular delivery of drugs, decrease the sensitivity to drugs as well as increase the tolerance to therapies.

These strategies of using inorganic nanocarriers to treat cancers demonstrate several advantages. The inorganic nanocarriers could extend the retention of drugs in blood circulation as they are quite stable, enhance drug accumulation in tumors, increase cellular uptake by cancer cells, help drugs to escape from the recognition of pump transporters in MDR cancer cells, and enhance the therapeutic efficacy of drugs. Besides, targeting ligands, drugs, imaging probes, and therapeutic genes/proteins could be easily loaded to inorganic nanocarriers to enhance the targeting ability, increase the bioavailability of drugs, achieve synergistic effects, and avoid degradation of bioactive compounds as well as unfavorable side effects. Moreover, inorganic nanocarriers serve as multifunctional platforms for drug/gene delivery, targeted/controlled release, and hyperthermia to overcome cancer MDR. Furthermore, the imaging function of inorganic nanocarriers can help to investigate the biological processes by molecular imaging, including the interaction between inorganic nanocarriers and cancer cells, identifying MDR related factors, tracing the pathway of drugs/nanocarriers inside cancer cells and tumor tissues, and monitoring therapeutic outcomes.^143^, ^144^

Compared to organic nanocarriers, the inorganic nanocarriers demonstrated several advantages, including easy preparation, easy size and morphology control, stable in aqueous solution, easy for surface modification and with imaging functions, such as gold nanoparticles and nanotubes. However, there are some concerns about inorganic nanocarriers for biomedical applications, including scale‐up preparation without quality change, modifying the compatibility, and potential nanotoxicity. It is crucial to conduct comprehensive studies of inorganic nanosystems in vitro and in vivo to obtain more effective and safe inorganic nanocarriers for clinical translation. The toxicity of each inorganic nanocarrier should be systemically studied, including acute and long‐term toxicity, harmfulness to normal tissues and organs, as well as teratogenicity. Besides, the pharmacokinetics and clearance of inorganic nanocarriers in the body should be considered while designing inorganic nanocarriers. More biocompatible inorganic nanocarriers could be achieved by modifying or hybrid with biocompatible polymers, peptides and other biomacromolecules. With further advances, multifunctional inorganic nanocarriers would be promising candidates for improving the efficacy of cancer treatment, especially for treating tolerable MDR cancers and providing cost‐effective omission of futile therapy.
